# Dual-Site Synergistic Modulation of La_0.9_Fe_0.8_Co_0.2_O_3_ Perovskite Oxide for High-Performance Methane Sensing in Oil and Gas Pipelines

**DOI:** 10.3390/s26196149

**Published:** 2026-09-28

**Authors:** Lichao Dong, Zhenzhu Xi, Lu Hu, Jiangang Zheng

**Affiliations:** 1 State Key Laboratory of Critical Minerals, School of Geosciences and Info-Physics, Central South University, Changsha 410083, China; dlc_cq@petrochina.com.cn; 2 No. 12 Oil Production Plant of Changqing Oilfield, Qingyang 745400, China; hul_cq@petrochina.com.cn (L.H.); zjig_cq@petrochina.com.cn (J.Z.)

**Keywords:** methane gas sensor, perovskite oxide, dual-site synergistic modulation, LaFeO_3_

## Abstract

Methane (CH_4_), the primary constituent of natural gas, is a colorless, odorless, combustible gas prevalent in petroleum and natural gas transmission pipelines as well as coal mines. However, conventional n-type semiconductor sensors face significant challenges in real-time CH_4_ leak monitoring within these environments due to severe signal drift induced by synergistic interference from high humidity and volatile organic compounds (VOCs). To address this, we synthesized La_0.9_Fe_0.8_Co_0.2_O_3_ nanomaterials via a facile one-pot hydrothermal method. A dual-site synergistic modulation strategy, involving the co-introduction of self-induced defects and Co dopants, endows the sensor with exceptional sensitivity toward CH_4_ at markedly lowered operating temperatures. At the optimal temperature of 125 °C, the tailored sensor exhibited a remarkable sensitivity of 17.55 toward 100 ppm CH_4_, representing an order-of-magnitude improvement over pristine LaFeO_3_. Furthermore, the device demonstrated excellent dynamic performance, with a rapid response time of 9.9 s and a recovery time of 7.2 s, enabling reliable detection across diverse environmental matrices. Notably, the sensor maintained a stable response value of approximately 15 even under extreme humidity (95% relative humidity) and exhibited outstanding repeatability. The exceptional performance originates predominantly from A-site-induced lattice strain coupled with the proliferation of active sites triggered by B-site substitution. The insights gained and the established mechanism herein enable the advancement of advanced methane sensing technologies geared towards industrial safety applications.

## 1. Introduction

As the cornerstone of the modern energy infrastructure, oil and gas resources rely heavily on long-distance buried pipelines for cross-regional allocation. However, pipeline integrity faces persistent threats from soil corrosion, geotechnical displacement, weld defects, and third-party interference, leading to frequent CH_4_ fugitive emissions. Unlike confined mine shafts, valve stations and urban gas utility corridors within transmission networks are typically semi-enclosed with dense ignition source distribution. Consequently, undetected minor leaks can accumulate in local pockets, forming explosive gas clouds reaching the lower explosive limit (LEL = 5% *v*/*v*) [1,2,3,4]. Upon contact with electrical sparks or electrostatic discharge, these mixtures pose imminent risks of jet fires or cascading explosions, resulting in catastrophic casualties, property damage, and exacerbated greenhouse effects. Therefore, developing methane sensing technologies that offer high sensitivity and rapid response while adapting to complex pipeline environments has become pivotal for advancing intelligent pipeline operation and maintenance (O&M), facilitating a strategic shift from reactive emergency response to proactive early warning systems [5,6,7,8].

In practical pipeline monitoring scenarios, methane sensors must not only accurately identify target gases but also effectively suppress interference from multifaceted environmental noise. Specifically, associated sour gases such as hydrogen sulfide (H_2_S) exhibit high toxicity and readily induce sulfur poisoning in sensing materials, causing irreversible device failure [5]. In high-humidity environments (e.g., coastal areas or flood seasons), water molecules aggressively compete with target gases for occupancy of active sites, leading to a marked degradation in sensing performance. Concurrently, the presence of ambient volatile organic compounds (VOCs) and substantial diurnal temperature variations impose stringent requirements on selectivity and long-term stability against zero-drift. Current mainstream detection technologies each present notable drawbacks: gas chromatography (GC) offers high accuracy but suffers from bulky instrumentation and labor-intensive maintenance, while wavelength-modulated diode laser spectroscopy enables rapid detection but remains prohibitively expensive for widespread deployment across extensive pipeline networks. In contrast, metal oxide semiconductor (MOS) sensors stand out as a highly prospective avenue for establishing comprehensive grid-based monitoring systems, owing to their miniaturization, low power consumption, seamless integration into Internet of Things (IoT) architectures, and significant cost advantages [9,10,11].

Among the diverse array of MOS sensing materials, ABO_3_-type perovskite oxides stand out due to their unique physicochemical properties. The prototypical p-type semiconductor LaFeO_3_ exhibits exceptional physicochemical stability and high redox activity in its surface lattice and adsorbed oxygen species. Crucially, the flexibility for heterovalent cation substitution at both the A and B sites allows for precise tailoring of band structures and surface active sites. Regrettably, conventional bulk LaFeO_3_ suffers from intrinsically low response values, a high optimal operating temperature (>200 °C), and sluggish response/recovery time, failing to meet the low-power and fast-response demands of field pipeline monitoring. Recent studies indicate that B-site doping with transition metals (e.g., Co, Cr, Ni) [12,13,14] effectively modulates carrier mobility and enhances oxygen vacancy concentration, thereby improving reducing gas sensitivity. This phenomenon primarily arises because transition metal dopants incorporate heterovalent cations into the crystal lattice, which disrupts local charge neutrality and promotes oxygen vacancy formation to preserve electrostatic equilibrium. Furthermore, the mixed valence states of these transition metals facilitate small-polaron conduction, thereby effectively tuning the carrier mobility. Parallel strategies involving A-site cation deficiency (i.e., creating A-site vacancies) disrupt lattice charge neutrality, inducing a higher population of surface-active oxygen species and optimizing microstructural morphology to reduce gas diffusion pathways.

Motivated by these imperatives, this study addresses the critical need for real-time pipeline monitoring by proposing a synergistic modification strategy that integrates A-site deficiency with B-site Co doping, leading to the successful synthesis of a novel sensing material, La_0.9_Fe_0.8_Co_0.2_O_3_. We systematically evaluated the gas-sensing performance of this material under simulated pipeline conditions. A combination of X-ray diffraction (XRD), N_2_ physisorption (BET), inductively coupled plasma mass spectrometry (ICP-MS), and scanning electron microscopy (SEM) was employed to thoroughly characterize the samples. This suite of techniques corroborated the successful formation of a pure perovskite phase and elucidated the doping-induced microstructural evolution, encompassing crystallinity, stoichiometry, surface area, and morphology. Evaluation of the gas-sensing properties reveals that, benefiting from the synergistic effect of A-site vacancies and Co substitution, the optimized sensor exhibits exceptional methane-sensing detection ability at a reduced working temperature of 125 °C. Especially, it achieves an excellent sensitivity of 17.55 toward 100 ppm CH_4_, coupled with rapid response (9.9 s) and recovery (7.2 s) times. Furthermore, the sensor maintains robust anti-humidity interference, long-term stability, and excellent batch-to-batch reproducibility under low-power operational parameters. Finally, combining material characterization with electrical analyses, we elucidate the enhancement mechanisms underlying improved surface oxygen chemisorption, charge transport behavior, and gas-sensing reaction kinetics. This work clarifies the methane detection mechanism in complex pipeline environments and provides a critical material foundation and theoretical framework for developing high-performance, low-power solid-state gas sensors for oil and gas stations, long-distance transmission pipelines, and urban gas distribution networks. The findings hold significant scientific importance and practical value for enhancing the intrinsic safety of energy transportation systems.

## 2. Experimental Section

### 2.1. Materials

Chemical reagents of analytical reagent (AR) grade were sourced from Aladdin Biochemical Technology Co., Ltd. (Shanghai, China) and were used without further purification. The synthesis employed lanthanum nitrate hexahydrate (La(NO_3_)_3_·6H_2_O, ≥99.9%, Aladdin Biochemical Technology Co., Ltd., Shanghai, China), iron(III) nitrate nonahydrate (Fe(NO_3_)_3_·9H_2_O, ≥99.5%, Aladdin Biochemical Technology Co., Ltd., Shanghai, China), and cobalt(II) nitrate hexahydrate (Co(NO_3_)_2_·6H_2_O, ≥99.9%, Aladdin Biochemical Technology Co., Ltd., Shanghai, China) as precursors, with anhydrous citric acid (C_6_H_8_O_7_, ≥99.9%, Aladdin Biochemical Technology Co., Ltd., Shanghai, China) introduced as a complexing agent to facilitate the hydrothermal reaction.

### 2.2. Synthesis of LaFeO_3_

The synthetic procedure commenced with the preparation of two distinct precursor solutions. Initially, 2.165 g of La(NO_3_)_3_·6H_2_O was dissolved in 20 mL of DI water and stirred magnetically at 75 °C for 0.5 h to ensure total solubilization. In parallel, 2.02 g of Fe(NO_3_)_3_·9H_2_O and 1.8 g of anhydrous citric acid were mixed in DI water under magnetic stirring at 85 °C for 50 min, promoting the chelation of metal ions. The resultant homogeneous solutions were then combined and subjected to hydrothermal crystallization in a Teflon-lined autoclave at 175 °C for 20 h. These elevated temperature and pressure conditions facilitated the in situ decomposition and structural rearrangement of the metal–citrate complexes, thereby nucleating the target perovskite framework. Post-reaction, the brownish colloidal suspension was isolated via centrifugation and subjected to a purification process, alternately rinsed three times with absolute ethanol and once with DI water, to remove residual impurities. The recovered solid was dried at a fixed temperature for 12 h, yielding a brown precursor powder. Finally, static-air calcination at 800 °C for 6 h, executed with a controlled heating ramp, ensured the complete removal of organic templates and the ultimate attainment of phase-pure LaFeO_3_.

### 2.3. Synthesis of La_0.9_Fe_0.8_Co_0.2_O_3_

The Co-substituted perovskite was synthesized via a similar hydrothermal route using identical high-purity reagents. The procedure mirrored that of the undoped sample but with adjusted stoichiometric ratios. Separate precursor solutions for the A-site and B-site cations were prepared. The A-site solution was prepared by dissolving 1.95 g of La(NO_3_)_3_·6H_2_O in 30 mL of DI water, followed by the addition of 0.9 g of anhydrous citric acid; the mixture was stirred magnetically at 60 °C for 20 min until homogeneous. Concurrently, the B-site solution was prepared by dissolving 1.616 g of Fe(NO_3_)_3_·9H_2_O, 0.1454 g of Co(NO_3_)_2_·6H_2_O, and 0.9 g of citric acid in 20 mL of DI water, maintained at 85 °C for 30 min to facilitate complete chelation of the transition metal ions. Upon cooling, these stoichiometric solutions were combined in a Teflon-lined autoclave. Hydrothermal processing at 175 °C for 20 h drove the co-precipitation and polycondensation reactions, leading to the formation of a La^3+^-Fe^3+^-Co^3+^ mixed-metal oxide framework. The resulting yellowish-brown slurry underwent a treatment analogous to that of the pristine sample, specifically, three cycles of centrifugation and washing with ethanol followed by a DI water rinse. Subsequent drying at 120 °C for 12 h yielded a yellowish-brown precursor powder. The final calcination step (800 °C, 6 h, static air) was pivotal: it ensured the exhaustive decomposition of residual nitrates/organics, promoted solid-state diffusion for homogeneous cation distribution, and ultimately crystallized the single-phase perovskite La_0.9_Fe_0.8_Co_0.2_O_3_ with a well-defined lattice. A schematic of the fabrication process is depicted in Figure 1.

### 2.4. Material Characterization

Correlative characterization was performed to elucidate the structural, compositional, and surface attributes of the synthesized specimens. Crystal phase identification was executed using an X-ray diffractometer (Ultima IV, Rigaku Corporation, Akishima, Japan), acquiring high-resolution patterns across a 2θ interval of 10–80°. Data acquisition parameters were optimized to maximize the signal-to-noise ratio, facilitating the precise assignment of diffraction peaks. Phase assemblages were determined by matching experimental patterns against entries in the JCPDS database, while crystallite dimensions were quantified via the Scherrer equation where appropriate. Complementing this, microstructural morphologies and particle size distributions were probed using field-emission scanning electron microscopy (FE-SEM, SU8600, Hitachi High-Tech Corporation, Tokyo, Japan). Imaging at various magnifications clearly revealed the surface topography and grain boundary structures. These images facilitated not only a qualitative assessment of the particle morphology but also a statistical analysis of the particle size distribution—a critical factor governing the effective specific surface area, which in turn influences performance in heterogeneous catalysis and gas-sensing applications. To quantify the specific surface area and pore structure, nitrogen adsorption–desorption isotherms were measured at 77 K using a physical adsorption analyzer (ASAP 2460, Micromeritics Instrument Corporation, Norcross, GA, USA). Nitrogen physisorption analysis provided the specific surface area via the Brunauer–Emmett–Teller (BET) method, while the pore size distribution was extrapolated from the desorption isotherm branch utilizing the Barrett–Joyner–Halenda (BJH) algorithm. These structural parameters provide crucial insights into the gas adsorption capacity of the materials. ICP-OES analysis (ICPE-9820, Shimadzu Corporation, Kyoto, Japan) afforded precise insights into the elemental constituents and stoichiometric integrity of the synthesized powders, ensuring the accuracy of target element ratios and confirming the chemical homogeneity of the perovskite oxides. Furthermore, electrochemical impedance spectroscopy (EIS, CHI760E, CH Instruments Corporation, Shanghai, China) was performed over a broad frequency range of 0.1 Hz to 100 kHz to investigate the electrical transport properties and interface impedance characteristics. EIS measurements were essential for understanding the charge transfer kinetics and conductivity of the synthesized materials. Finally, the functional performance of these perovskites as chemiresistive sensors was evaluated using a commercial CGS-8 gas-sensing test system. This setup enabled the precise monitoring of dynamic response–recovery curves under controlled atmospheres, thereby directly correlating the materials’ structural attributes with their gas-sensing performance.

### 2.5. Sensor Preparation and Gas-Sensing Evaluation

The gas sensors were fabricated on commercial ceramic tube platforms pre-equipped with interdigitated Au–Pt electrodes. Initially, 15 mg of the as-synthesized sensing material was thoroughly ground in an agate mortar to ensure homogeneity. To prepare a viscous, printable paste, a mixed solvent system consisting of 5 μL of absolute ethanol and 10 μL of deionized water was added dropwise to the powder, followed by continuous grinding until a uniformly dispersed slurry was obtained. Subsequently, the slurry was loaded into a custom spray nozzle and deposited onto the ceramic tube surface via aerosol jet coating at a constant pneumatic pressure of 2 N. This technique ensured uniform coverage of the electrode gaps, establishing reliable conductive pathways. Following deposition, the sensors underwent a thermal curing step, being baked in a convection oven at 60 °C for 12 h. This low-temperature annealing process served a dual purpose: evaporating residual solvents and enhancing interfacial adhesion between the sensing film and the ceramic substrate, thereby forming a mechanically stable and chemically inert sensing layer. The ceramic tube was then carefully soldered onto a standard sensor base. A Nichrome heating wire was threaded through the central bore of the tube and secured to the base. This internal heater functioned as a precision temperature regulator, allowing for accurate control and maintenance of the operating temperature during gas exposure tests. To stabilize the electrical baseline and eliminate residual adsorbates or volatile impurities from synthesis, the assembled sensors were aged on a dedicated aging station prior to formal testing. All target gases used in this study were sourced from certified gas cylinders with guaranteed purities of ≥99.99%. The gas-sensing performance was quantitatively evaluated by defining the sensor response (S) differently for reducing and oxidizing analytes to reflect distinct carrier modulation mechanisms. For typical reducing gases (e.g., CH_4_, H_2_S, and NH_3_), the response was formulated as the quotient of the resistance in the target gas (R_g_) to that in dry air (R_a_), as expressed in Equation (1) [15]:

S = R_g_/R_a_ (1)

Conversely, for oxidizing gases (e.g., NO_2_, NO), the response was formulated as the inverse quotient to maintain consistency in performance interpretation, as shown in Equation (2) [16]:

S = R_a_/R_g_ (2)

Other experimental conditions and environmental parameters (e.g., relative humidity, flow rates) were kept consistent for both gas categories. Furthermore, the transient dynamic characteristics of the sensors were characterized by two key parameters: response time and recovery time. Kinetic analysis of the sensing behavior defined the response dynamics. Specifically, the response time quantified the kinetics of reaching 90% of the equilibrium resistance in the target gas. Conversely, the recovery time characterized the desorption kinetics, measured as the time required for 90% restoration of the initial baseline resistance following gas cutoff and subsequent purging with dry air [17,18,19,20]. Specifically, LFO denotes LaFeO_3_, while LFCO denotes La_0.9_Fe_0.8_Co_0.2_O_3_.

## 3. Results and Discussion

### 3.1. Structural Characteristics and Analysis

Phase identification via XRD, as documented in Figure 2a, confirms the successful synthesis of both LFO and LFCO. Comparison with the standard card for orthorhombic LaFeO_3_ (PDF#37-1493, space group Pnma) [21,22] indicates that the characteristic diffraction peaks of both samples correspond precisely to the reference data, with no detectable impurity peaks. This confirms that the as-prepared powders possess a single and pure perovskite phase. The results indicate that the introduction of A-site deficiencies and B-site Fe/Co co-doping did not disrupt the host lattice framework; instead, the dopant ions successfully accommodated within the lattice sites, yielding a stable solid solution. Furthermore, the sharp diffraction peaks and high signal-to-noise ratio reflect the excellent crystallinity of the samples, laying the groundwork for investigating doping-induced lattice distortions and their regulatory mechanisms on gas-sensing performance [23]. Figure S1 displays the XPS spectra of La_0.9_Fe_0.8_Co_0.2_O_3_. The La 3d spectrum (Figure S1a) exhibits two characteristic doublets at 834.58/838.39 eV and 851.14/855.59 eV, which are assigned to the La 3d3/2 and La 3d5/2 spin states, respectively, indicating the presence of La^3+^. In the Fe 2p XPS spectrum (Figure S1b), peaks observed at 710.15/712.03 eV and 723.3/724.93 eV are attributed to Fe 2p3/2 and Fe 2p1/2, further confirming the trivalent oxidation state of iron. Additionally, the high-resolution Co 2p spectrum (Figure S1c) shows two prominent peaks at 780.79 eV and 787.04 eV, corresponding to Co 2p3/2 and Co 2p1/2, which provides solid evidence for the successful incorporation of Co^3+^ [12].

The structural evolution prompted by Co doping is quantified in Table 1. Relative to LFO, the LFCO lattice undergoes significant dimensional perturbations, characterized by a uniform attenuation of the a-, b-, and c-axes. This collective compression results in a substantially diminished unit cell volume, underscoring the profound steric effects of substituting Fe^3+^ with smaller Co^3+^ cations. On one hand, the reduction of the A-site La3+ stoichiometry to 0.9 induces A-site vacancies (VA), weakening the lattice support originally provided by the large lanthanum ions and decreasing the Goldschmidt tolerance factor. On the other hand, at the B-site, partial substitution occurs where Fe^3+^ ions (high-spin, 0.645 Å for CN = 6) are exchanged for smaller Co^3+^ ions (high-spin, 0.61 Å) [24,25,26]. Although Co^2+^ (0.745 Å) generally exhibits poor stability in perovskite structures, Co^3+^ predominates in this study; its smaller effective ionic radius further exacerbates the lattice compression effect. The synergistic effect arising from structural relaxation induced by A-site vacancies and chemical pressure generated by small-radius B-site doping forces a uniform contraction of the orthorhombic lattice. This variation strictly adheres to Vegard’s law, wherein the lattice parameters of a solid solution decrease linearly with the average radius of the solute ions. The reliability of the Rietveld refinement and the significant reduction in unit cell volume not only confirm the successful incorporation of Co^3+^ into the B-site of the perovskite lattice at the microscopic level but also provide conclusive evidence for the successful synthesis of the target compound La_0.9_Fe_0.8_Co_0.2_O_3_.

To quantitatively elucidate the doping-induced lattice evolution, Rietveld refinement of the LFCO XRD data was performed using GSAS-II v5.6.0 software. As illustrated in Figure 2b, the refined curve aligns exceptionally well with the experimental data points, exhibiting minimal fluctuations in the difference line, which indicates a reasonable structural model. The refinement converged satisfactorily, yielding a goodness-of-fit indicator (χ^2^) of 3.231 and a weighted profile R-factor (Rwp) of 4.11%. These parameters fall within the universally accepted ranges for perovskite oxide structure refinements, robustly confirming the reliability of the obtained crystallographic information, such as unit cell parameters and atomic coordinates. This provides a solid data foundation for the in-depth discussion of microstructural distortions triggered by the synergistic A/B-site strategy.

The representative FE-SEM images rendered in Figure 3 afford a direct visualization of the microstructural modifications triggered by doping. In contrast to the undoped LFO sample, which primarily comprises irregular bulk particles characterized by severe hard agglomeration, a dense structure, and a paucity of pores—factors that inherently restrict gas diffusion pathways—the synergistically modified LFCO sample exhibits a striking morphological reconstruction. The LFCO particles are significantly refined, display a more uniform size distribution, and spontaneously assemble into well-defined microspherical aggregates. This distinctive micro–nano architecture is primarily attributed to lattice distortions and surface energy redistribution triggered by A-site deficiencies and B-site doping. The introduction of heteroions disrupts the periodic potential field of the lattice, suppressing abnormal grain growth at elevated temperatures while simultaneously inducing the oriented assembly of primary nanoparticles to construct a porous network. This microstructure, composed of stacked nanoscale primary particles, not only effectively inhibits excessive grain growth and agglomeration but also substantially increases surface roughness and the effective specific surface area. From a gas-sensing perspective, this optimized morphology significantly shortens gas diffusion pathways and exposes a greater number of intrinsic active sites. It thereby furnishes an ideal physical venue for the surface adsorption of oxygen and target gases, as well as subsequent catalytic oxidation reactions, fundamentally laying the structural groundwork for the superior gas-sensing performance of LFCO [27,28].

Nitrogen physisorption analysis, the results of which are plotted in Figure 4, reveals the textural properties and pore size distributions of the two samples. Quantitative analysis reveals a dramatic enhancement in the specific surface area of the LFCO sample (25.95 m^2^/g) compared to its pristine LFO counterpart (4.17 m^2^/g)—an increase of over sixfold. This significant microstructural optimization is mainly ascribed to a doping-induced pinning effect. On one hand, self-induced defects elevate the defect concentration within the lattice, effectively suppressing grain boundary migration and excessive grain growth during high-temperature calcination. On the other hand, lattice distortions induced by doping alter the system’s surface energy, thereby modulating nucleation kinetics and mitigating particle agglomeration. Consequently, LFCO develops a porous network structure formed by the stacking of nanoscale primary particles, which drastically shortens gas diffusion pathways. Regarding the gas-sensing mechanism, the exponential increase in specific surface area directly translates to a higher density of surface active sites. This not only provides a vast platform for gas molecule adsorption but also significantly enhances the surface reactivity of the material. Furthermore, the capillary condensation effect fostered by high porosity facilitates the permeation and diffusion of target gases into the sensing layer, enabling more charge carriers to participate in surface redox reactions. Macroscopically, this manifests as an elevated response value and accelerated response/recovery time. In summary, the A-site defect engineering strategy achieves a dual optimization of specific surface area and electronic transport properties by regulating the crystallographic behavior and microstructure [29,30]. This improvement in intrinsic material properties exhibits a high degree of structure–property correlation with the aforementioned gas-sensing results, strongly validating the scientific efficacy of this modification strategy in overcoming the performance bottlenecks of conventional sensing materials.

To further elucidate the charge transport behavior within the materials, electrochemical impedance spectroscopy (EIS) was employed to characterize the electrical conductivity of the samples. Figure 5 displays the Nyquist plots acquired at various temperatures, where the semicircular capacitive arcs in the high-frequency region visually reflect the complex impedance characteristics. Evidently, the radii of these arcs for both samples contract regularly with increasing temperature, conforming to the physical principle that thermal excitation increases charge-carrier concentration in semiconductors. Notably, within the same temperature range, the LFCO sample exhibits significantly smaller arc radii than the pristine LFO, unequivocally demonstrating a substantial reduction in intrinsic impedance and confirming the tangible optimization of charge transfer dynamics by the doping strategy. From a microscopic standpoint, the introduction of A-site cation vacancies and heterovalent doping effectively modulated the band structure, lowering the activation energy required for carrier hopping. This synergistic effect not only augmented the concentration of free charge carriers but also diminished the height of potential barriers formed by space charge regions at grain boundaries, thereby markedly enhancing electron mobility. The low-impedance characteristics revealed by EIS directly bolster the efficiency of charge exchange during gas-sensing reactions: lower impedance implies a more rapid electron transfer process between surface-adsorbed oxygen species and target gas molecules. This, in turn, significantly shortens the sensor response/recovery times and reinforces the stability of signal transduction. Collectively, the excellent dielectric response and charge transfer kinetics of LFCO establish a robust electrical foundation for its manifestation of exceptional sensitivity coupled with accelerated response kinetics in gas-sensing applications [31,32].

Figure 6 delineates the evolution of sensing responses for both LFO and LFCO sensors across a broad temperature spectrum (100–300 °C). A pivotal observation is that LFCO exhibits an initial response threshold at 125—significantly lower than that of LFO—indicating that the dual-site synergistic modification activates surface reactivity at substantially reduced thermal energy. More strikingly, at their respective peak performance temperatures, the response intensity of LFCO surpasses that of LFO by a factor of five, accompanied by a drastic OOT reduction of approximately 175 °C. This desirable low-temperature, high-response paradigm is mechanistically linked to the electronic structure remodeling and surface chemical state optimization induced by A-site defects. Firstly, the introduced defect energy levels lower the activation barrier for surface reactions, inherently boosting catalytic activity. Secondly, the elevated carrier concentration facilitated by doping accelerates the dissociation of surface-adsorbed oxygen species, thereby minimizing the energy expenditure required for gas detection. It is noteworthy that beyond the optimal point of 125 °C, the response of LFCO follows a characteristic “volcano-shaped” trend, peaking before declining sharply at higher temperatures (up to 300 °C). This attenuation is ascribed to the thermodynamic reversal of physicochemical adsorption equilibria; excessive thermal energy exponentially accelerates gas desorption rates, overwhelming adsorption kinetics and preventing the maintenance of sufficient surface coverage on active sites [33]. Consequently, these temperature-dependence profiles precisely define the optimal operational window as the dynamic equilibrium point between adsorption and desorption kinetics. In essence, these findings robustly validate the efficacy of the synergistic doping strategy. By dramatically lowering power consumption while enhancing sensitivity, LFCO effectively transcends the high-temperature bottleneck endemic to conventional perovskite sensors, heralding significant potential for low-power IoT applications.

Figure 7 systematically characterizes the dynamic sensing behaviors of the LFO and LFCO sensors upon exposure to varying concentrations of methane. As depicted in Figure 7a, both sensors exhibit a monotonic increase in response values with rising CH_4_ concentrations (1–100 ppm), with LFCO maintaining a substantially higher signal intensity throughout. Specifically, at the OOT of 125 °C, LFCO registers response values of 2.4, 3.25, 4.51, 6.01, 10.45, and 17.55 corresponding to CH_4_ concentrations of 1, 5, 10, 25, 50, and 100 ppm, respectively. Further quantitative comparison of the transient response–recovery curves (Figure 7b,c) highlights the kinetic superiority of LFCO. While LFO exhibits relatively sluggish response–recovery times of 17.1 s/10.8 s, the synergistically doped LFCO demonstrates a significant acceleration to 9.9 s and 7.2 s, respectively. This kinetic enhancement is directly attributable to the enlarged specific surface area and improved electrical conductivity, which facilitate faster adsorption–desorption kinetics and more efficient interfacial charge transfer. To evaluate the practical utility of the sensor, linear regression analysis was performed on the relationship between response and concentration (Figure 7d). The results reveal exceptional linearity across the entire tested concentration range (1–100 ppm). A single linear fit yields y = 0.15137x + 2.54318 (R^2^ = 0.996), demonstrating a highly reliable and predictable response. This excellent linear relationship across both low- and high-concentration regimes underscores the reliability of the sensor output.

Figure 8 presents a holistic evaluation of the LFCO sensor’s performance metrics pertinent to practical deployment. Initially, selectivity tests against common interfering gases (Figure 8a) reveal that at 100 ppm, the LFCO sensor demonstrates a markedly superior response magnitude to CH_4_ compared to ethanol, ethylene glycol, NO_2_, NH_3_, and CO_2_ (all tested interfering gases were maintained at a concentration of 100 ppm). Specifically, the response to CH_4_ is approximately sevenfold that of CO_2_. This exceptional discrimination capability is rooted in the specific catalytic oxidation pathways available on LFCO surfaces and the tailored surface chemistry that favors CH_4_ interaction. Subsequently, the influence of ambient humidity—a notorious factor for MOS sensor drift—was investigated (Figure 8b). Employing saturated salt solutions to generate a gradient from 35% to 95% relative humidity (RH), we observed a segmented decline in response, consistent with the competitive adsorption of H_2_O molecules occupying active sites. Crucially, even under extreme humidity (95% RH), the sensor retained approximately 78% of its initial response value, demonstrating robust moisture resilience. Regression analysis further delineated two linear regimes, namely y = −0.13595x + 22.5251 (low humidity, <54% RH) and y = −0.043x + 17.85062 (high humidity, >75% RH), indicating predictable performance across diverse climatic conditions. Furthermore, dynamic cycling tests (Figure 8c) affirmed the sensor’s reliability; over five consecutive cycles, the response–recovery curves remained highly reproducible with negligible baseline drift, reflecting the structural robustness imparted by the doping strategy. Long-term stability assessments (Figure 8d) corroborated these findings, showing that both baseline resistance and response values fluctuated within a narrow margin of ±2% over 30 days, with minor variations attributable to diurnal lab environment shifts. Collectively, the LFCO sensor integrates high selectivity, strong moisture resistance, excellent reversibility, and outstanding long-term stability, conclusively demonstrating its immense potential.

### 3.2. Gas-Sensing Mechanism

Building upon the widely accepted oxygen ion adsorption theory, this study provides a comprehensive mechanistic elucidation of the methane (CH_4_) sensing properties of the dual-site doped LFCO perovskite. As a prototypical reducing gas, CH_4_ participates in redox processes with surface-adsorbed oxygen anions on the sensing layer, which constitutes the fundamental origin of the systematic variations in macroscopic electrical signals. Integrated material characterization and gas-sensing measurements confirm that the LFCO material exhibits characteristic p-type semiconducting behavior, wherein electrical conduction is predominantly governed by holes. The sensing process can be delineated into two dynamically evolving stages: the equilibration phase in ambient air and the reaction phase upon CH_4_ exposure.

In ambient atmospheres, dioxygen molecules undergo surface chemisorption, extracting electrons from the valence band of the p-type semiconductor. This process reinforces the hole-accumulation layer via upward band bending, priming the material for analyte interaction. This process facilitates their conversion into highly reactive chemisorbed oxygen species (O_2_^−^, O^−^, or O^2−^) [34]. The distribution of these species is thermodynamically modulated by the operating temperature; while O_2_^−^ predominates at low temperatures (<100 °C), O- emerges as the dominant species at the optimal operating temperature of 125 °C due to its superior thermodynamic stability and reactivity. This electron-withdrawing process depletes the near-surface electronic density, resulting in the expansion of the hole-accumulation layer (HAL) or, concomitantly, the near-surface space-charge region undergoes progressive widening, elevating the interfacial potential barrier for hole transport. For p-type semiconductors, the resultant elevation in near-surface hole concentration significantly reduces the material’s resistivity, establishing the baseline resistance (R_a_). Upon CH_4_ exposure, the pre-chemisorbed electrophilic oxygen species instigate the oxidative dehydrogenation of methane, catalyzing the oxidation of methane molecules into CO_2_ and H_2_O and thereby consuming the surface-adsorbed oxygen species. This heterogeneous catalytic cycle culminates in the re-injection of the formerly entrapped electrons into the valence band of the p-type semiconductor. Upon re-exposure to ambient air, O_2_ molecules rapidly reoccupy the active sites vacated by the methane reaction and extract electrons from the valence band of the p-type semiconductor to regenerate chemisorbed oxygen species. This process not only facilitates the recovery of the HAL but also promotes the re-expansion of the near-surface charge region, ultimately driving the electrical resistance back to its initial baseline. Consequently, the LFCO sensor exhibits excellent repeatability and fast response/recovery time, which are attributed to this continuous and reversible cycle involving ambient oxygen chemisorption, redox reactions with methane, and subsequent re-oxidation. The relevant surface reaction kinetics are summarized as follows [35]:

O_2_(gas) → O_2_(ads) (3)

O_2_(ads) + 2e^−^ → 2O^−^(ads) (4)

CH_4_ + 4O^−^ → CO_2_ + 2H_2_O + 4e^−^ (5)

In the context of p-type semiconductors, these released electrons recombine with the excess surface holes, leading to a reduction in hole concentration, shrinkage of the HAL, and concomitant broadening of the EDL. Macroscopically, this manifests as a significant increase in electrical resistance (R_g_). Adhering to the conventional definition for p-type semiconductors, the sensor response (S) is defined as R_g_/R_a_, where the magnitude of this resistance change correlates positively with the CH_4_ concentration.

The exceptional gas-sensing performance of the LFCO sensor originates from the synergy between this surface mechanism and the structural modifications induced by the A-/B-site co-doping strategy. X-ray diffraction (XRD) analysis unequivocally confirms the effective solid solution of the dopants and the successful synthesis of phase-pure LFCO. Notably, electrochemical impedance spectroscopy reveals a significant enhancement in charge transport capabilities across both the bulk and grain boundaries in the dual-site doped material, which serves as the physical cornerstone for the improved sensing properties. The underlying mechanisms can be systematically elucidated as follows. Firstly, the B-site Fe/Co co-doping acts as the core driving force for constructing an efficient charge transport network. As typical transition metals, Fe and Co exhibit mixed valency (e.g., Fe^3+^/Fe^4+^ and Co^2+^/Co^3+^) within the lattice [36,37]. This valence diversity fulfills the prerequisite for the small polaron conduction mechanism, facilitating electron hopping between adjacent metal ion sites and establishing microscopic conductive pathways within the crystal lattice. Secondly, the introduction of A-site deficiencies induces pronounced lattice distortion and internal stress. This micro-strain field modulates the stereochemistry of the Fe/Co-O coordination spheres, optimizing the geometric configuration for electron hopping and thereby significantly lowering the activation energy barrier for electron migration. Concurrently, the A-site vacancies disrupt the original charge balance. Working in concert with the B-site doping effect, they modulate the formation energy and spatial distribution of oxygen vacancies, further optimizing the carrier concentration and creating a localized chemical environment conducive to electron transport. Finally, the dual-defect strategy effectively suppresses the tendency toward long-range cationic ordering in the lattice, preventing the formation of scattering centers arising from charge ordering or cluster effects. This atomic-scale disorder promotes the homogeneous dispersion of multivalent ions, thereby constructing a continuous, interconnected, and uniform percolation network throughout the material matrix [38,39]. This network drastically mitigates the scattering probability and interfacial potential barriers during long-range electron transport, leading to an order-of-magnitude enhancement in overall electrical conductivity. In summary, a significant synergistic gain effect arises from the combination of A-site defects and B-site doping. B-site doping serves to populate the lattice with abundant hopping sites, laying the groundwork for efficient electron transport. Concurrently, A-site engineering mitigates the energetic barriers for charge transfer and optimizes carrier statistics. Collectively, these modifications forge a highly efficient 3D conductive architecture, thereby accelerating the charge exchange dynamics at the gas–solid interface [40,41,42,43]. This mechanistic insight not only elucidates the enhanced performance of the LFCO sensor but also offers a robust blueprint for the rational design of high-performance semiconducting sensing platforms.

## 4. Conclusions

Harnessing the synergistic potential of A-site cation deficiency and B-site cobalt doping, this study successfully constructed a novel LaFe_1−x_Co_x_O_3_ (LFCO) perovskite architecture via a facile hydrothermal route, targeting high-efficiency methane detection. The resultant sensor exhibits exceptional chemiresistive performance at a substantially reduced optimal operating temperature of 125 °C, delivering a salient response magnitude of 17.55 towards 100 ppm CH_4_. Mechanistic investigations reveal that the A-site vacancies optimize the local electronic structure while promoting oxygen vacancy formation, and the incorporated Co species significantly enhance the redox activity and gas diffusion kinetics. Consequently, the device not only affords high sensitivity but also features ultrafast response/recovery rates, superior selectivity against prevalent interfering gases, and remarkable long-term endurance under harsh conditions, particularly high relative humidity. These results firmly position LFCO as a leading functional material for the upcoming generation of IoT-based environmental monitoring nodes. Furthermore, this work elucidates a universal material genome approach for engineering multi-element doped oxides, paving the way for the rational design of next-generation semiconductor gas sensors.

## Figures and Tables

**Figure 1:**
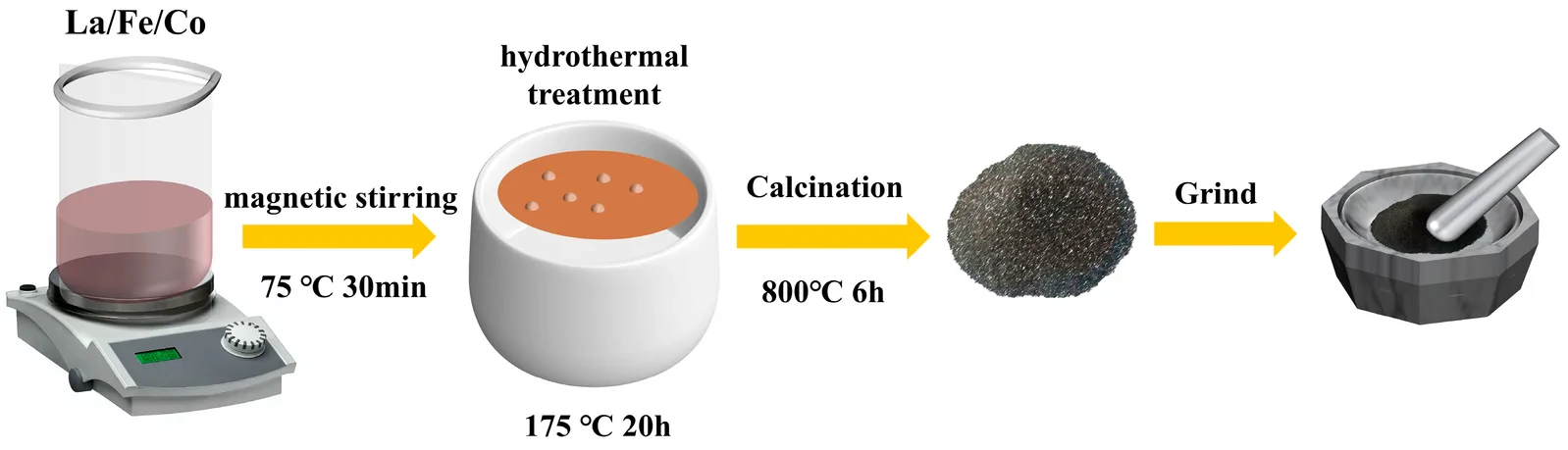
The flowchart of La_0.9_Fe_0.8_Co_0.2_O_3_ (illustration created by the author).

**Figure 2:**
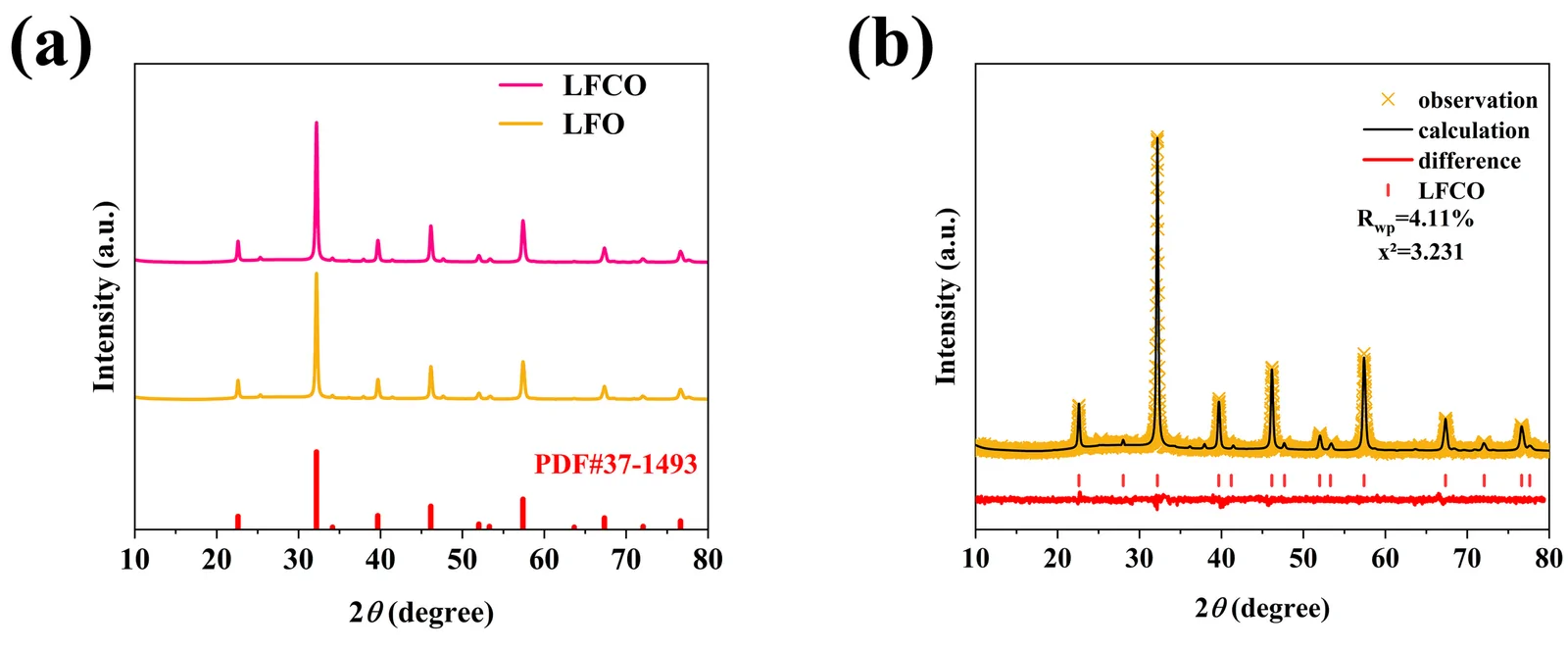
XRD patterns of (**a**) LFO and LFCO; (**b**) XRD refinement results of LFCO.

**Figure 3:**
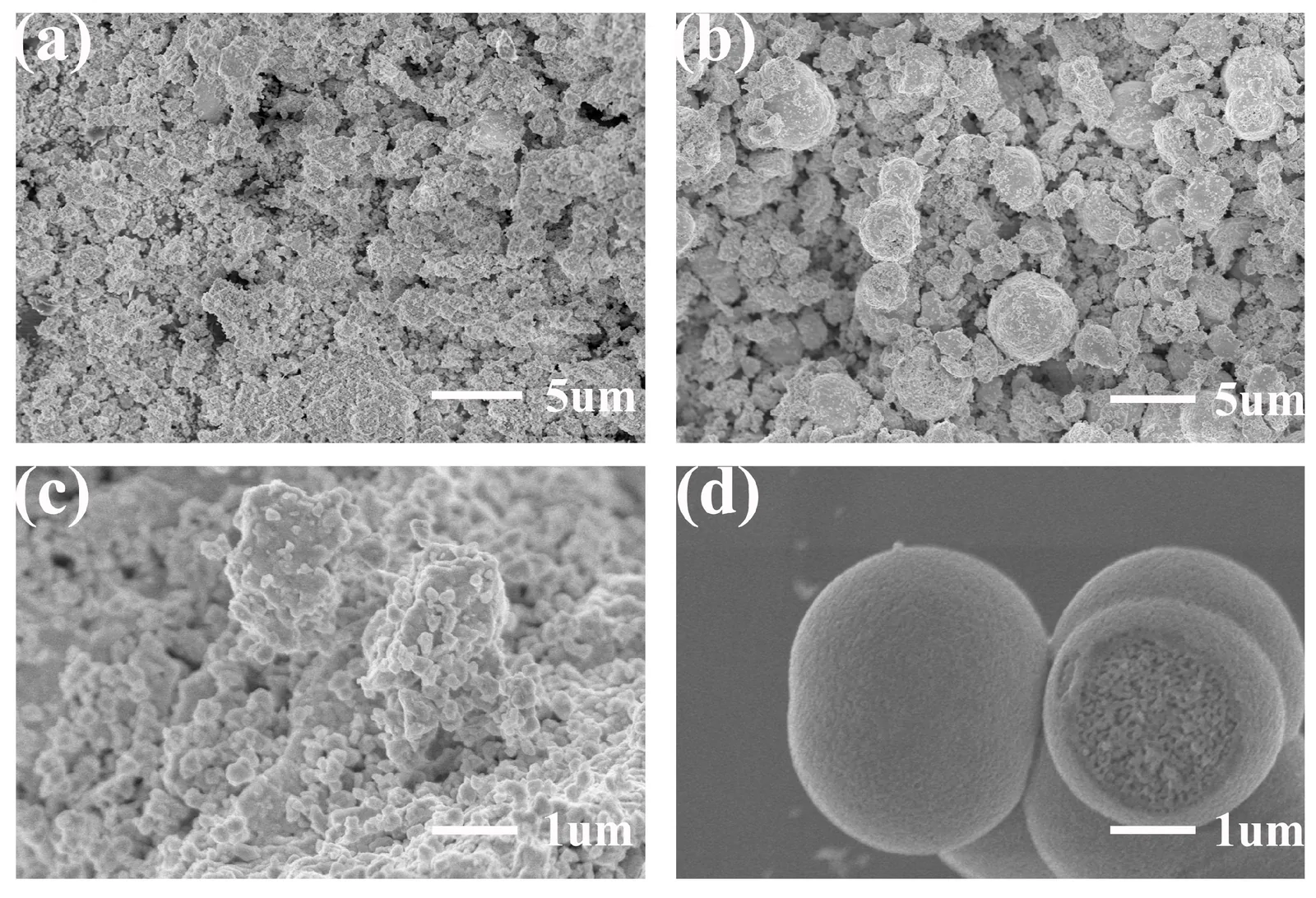
SEM morphological features of (**a**,**c**) LFO and (**b**,**d**) LFCO.

**Figure 4:**
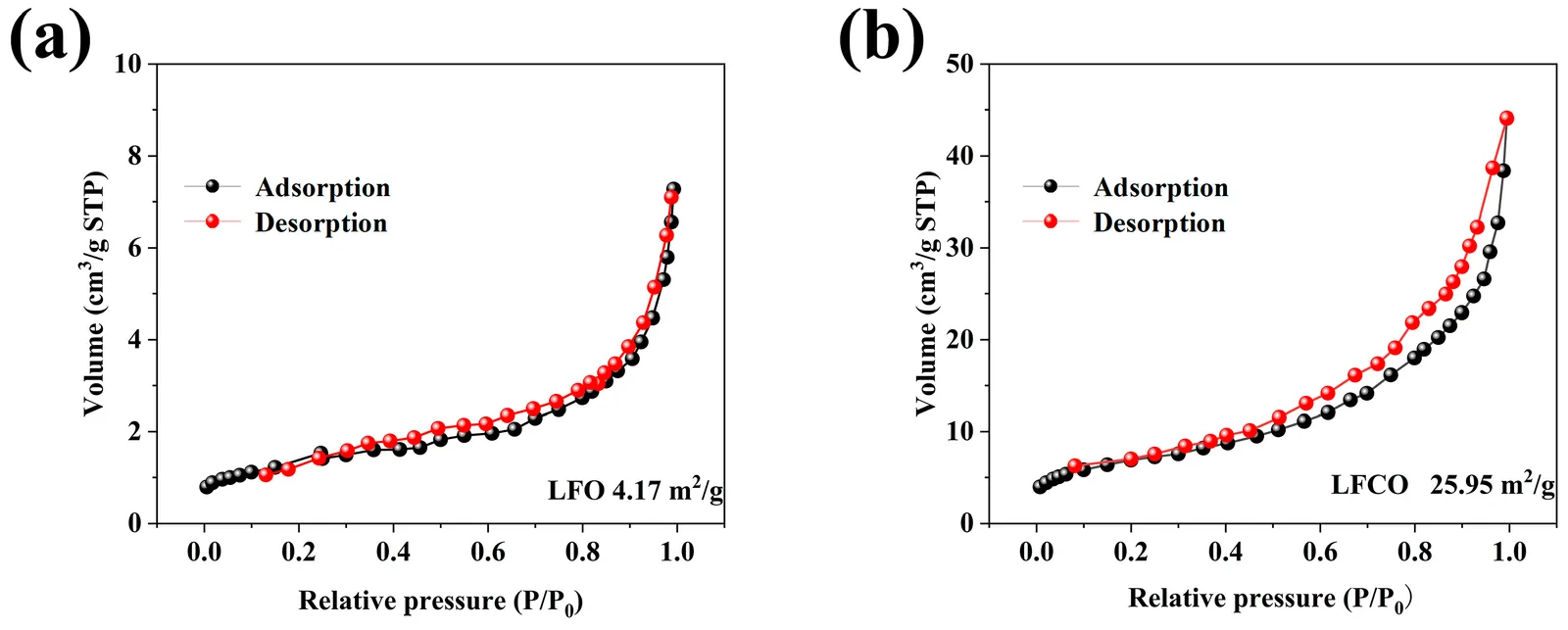
N_2_ adsorption–desorption isotherms: (**a**) LFO; (**b**) LFCO.

**Figure 5:**
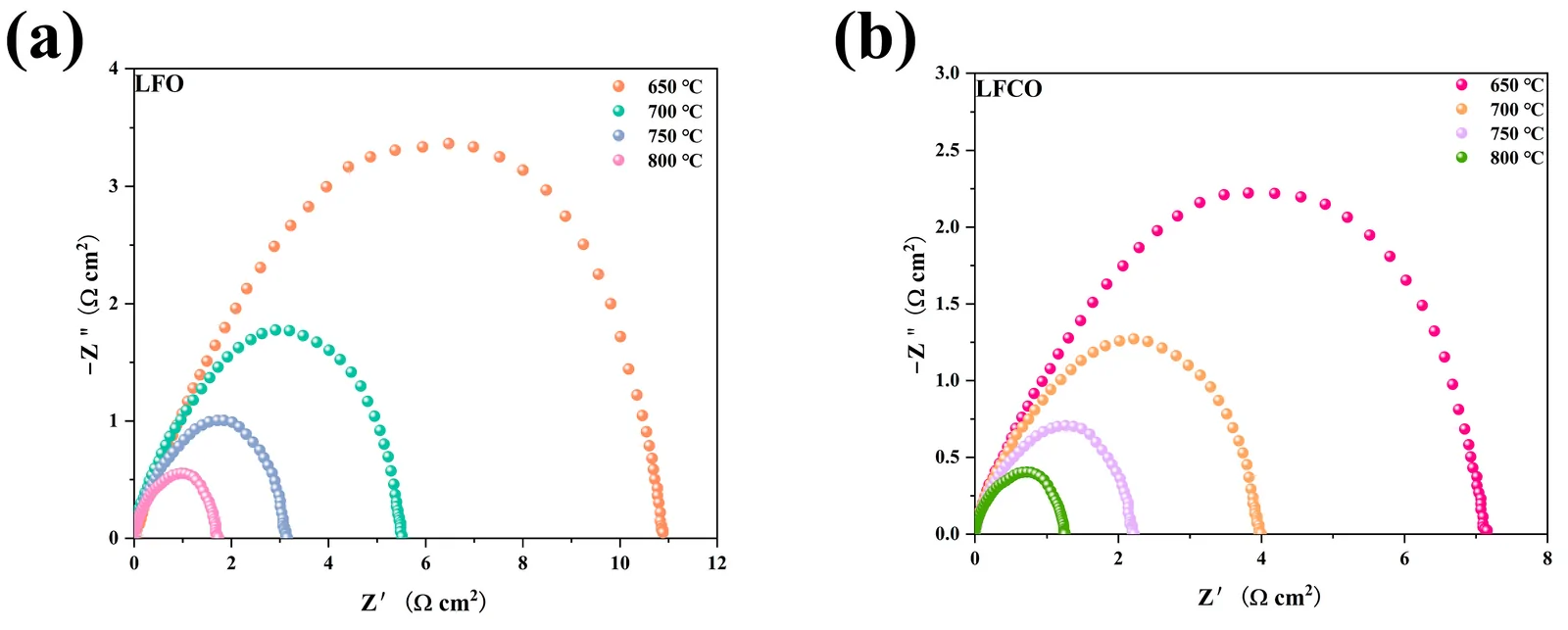
EIS curves at different temperatures: (**a**) LFO; (**b**) LFCO.

**Figure 6:**
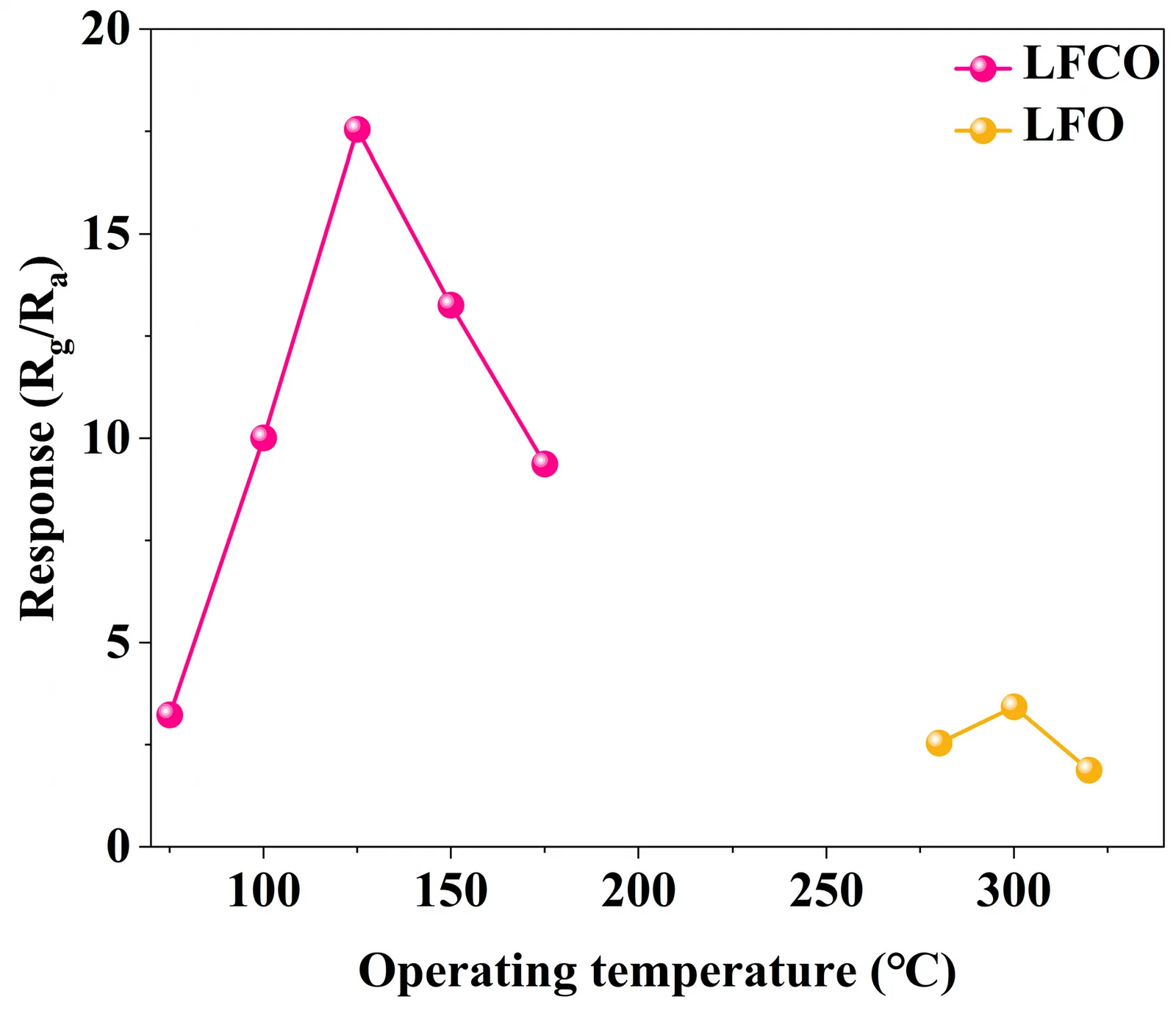
Different response values of two sensors at different working temperatures.

**Figure 7:**
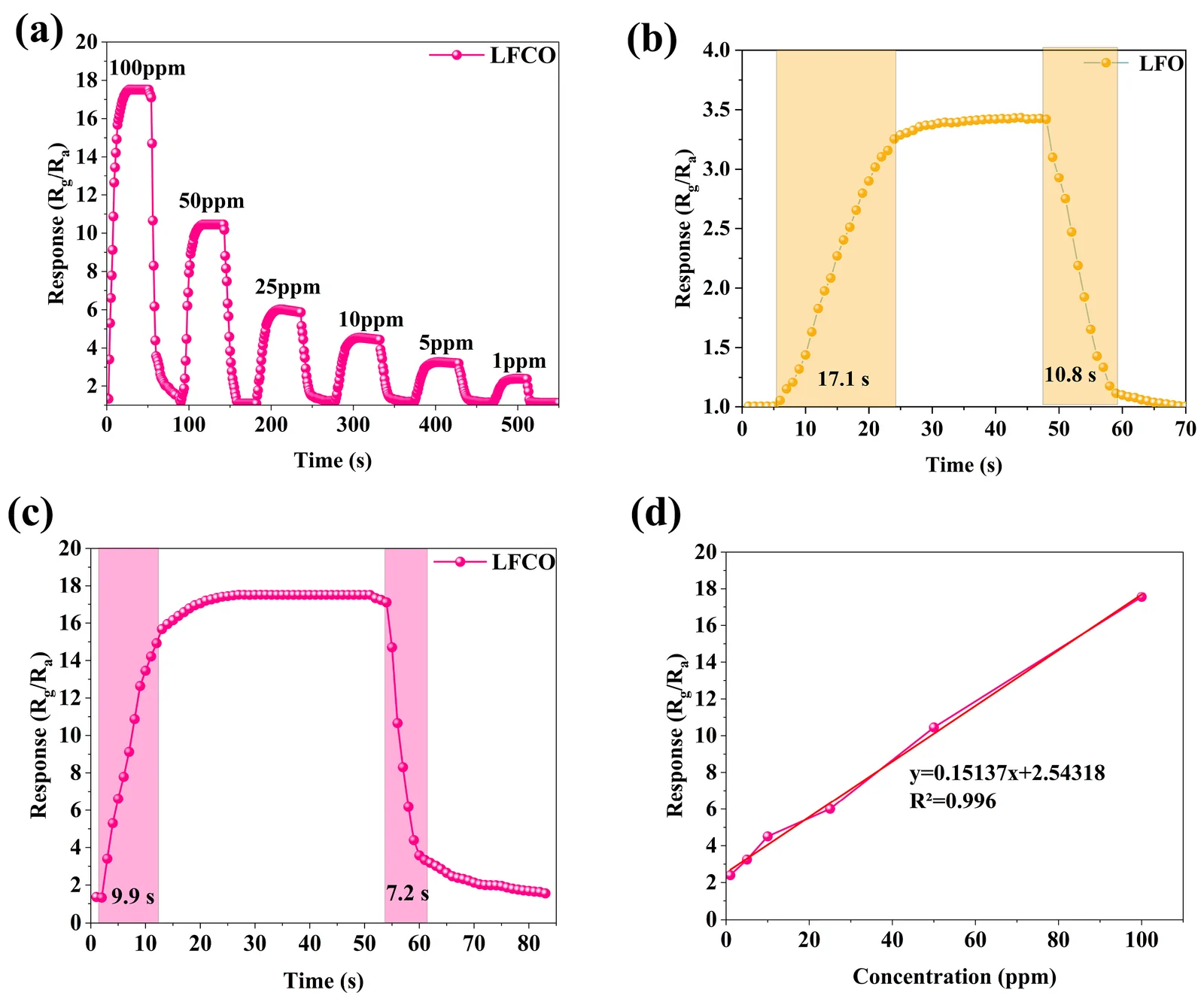
(**a**) Concentration-dependent curves of LFCO from 1 to 100 ppm; (**b**) dynamic response and recovery curve of LFCO; (**c**) dynamic response and recovery curve of LFO; (**d**) fitted response values of LFCO as a function of concentration from 1 to 100 ppm.

**Figure 8:**
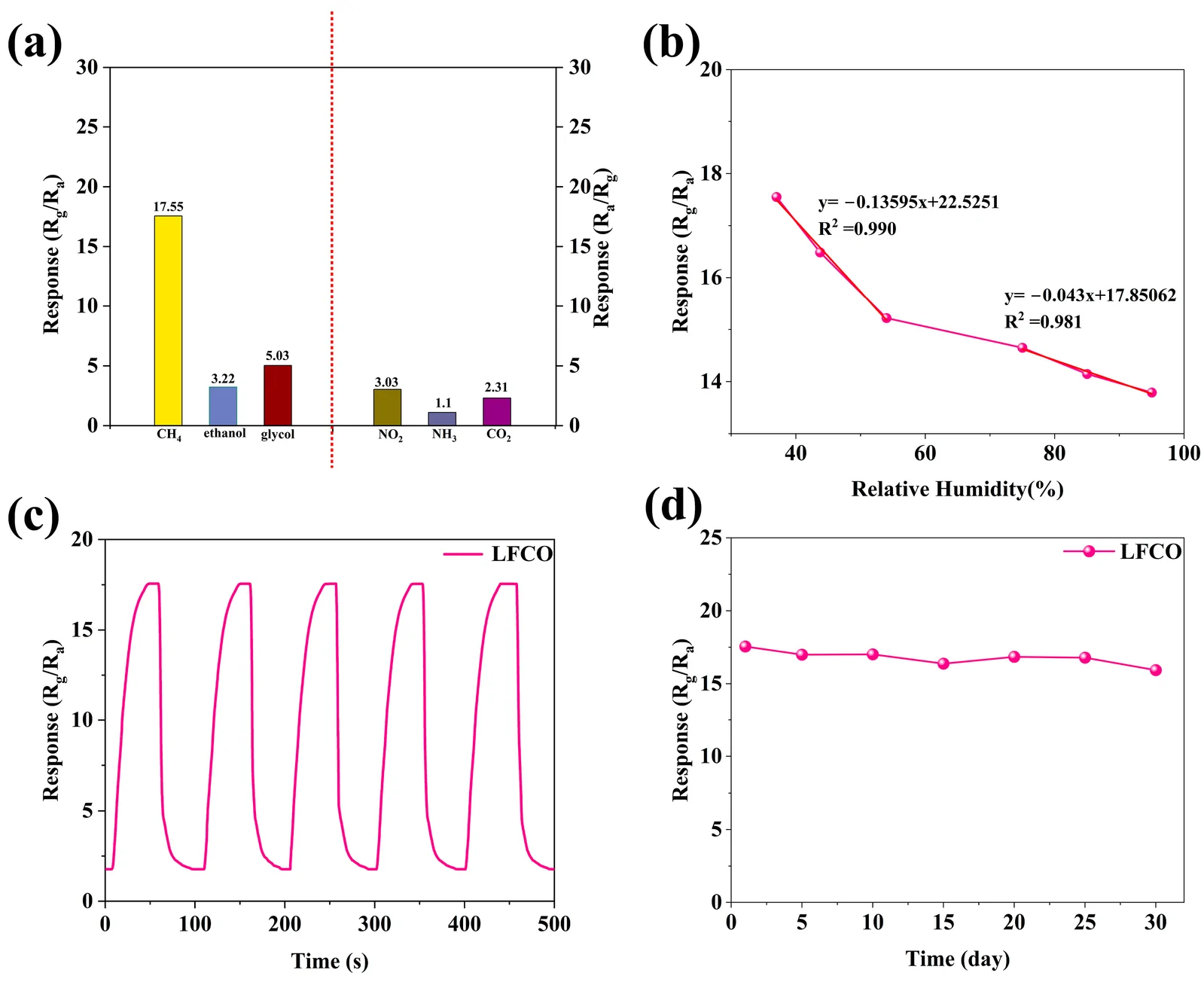
(**a**) Response of LFCO to different gases; (**b**) fitted curve of the response of LFCO under different humidity levels; (**c**) repeatability curve of LFCO; (**d**) long-term stability test of LFCO.

**Table 1:** Refined Unit cell parameters of LFO and LFCO.

Different Samples	Crystallite Size (nm)	Lattice Parameters (Å)			Lattice Volume (Å^3^)
		a	b	c	
LFO	29.671	5.550	7.853	5.550	242.1
LFCO	28.46	5.528	7.839	5.541	241.33

## Data Availability

The original contributions presented in this study are included in the article. Further inquiries can be directed to the corresponding author.

## Supplementary Materials

The following supporting information can be downloaded at https://www.mdpi.com/article/10.3390/s26196149/s1. References [44,45,46,47,48,49] are cited in the Supplementary Materials.
