# Supplementary material for: Dual-Site Synergistic Modulation of La0.9Fe0.8Co0.2O3 Perovskite Oxide for High-Performance Methane Sensing in Oil and Gas Pipelines
Source: Sensors (Basel). 2026 Sep 28;26(19):6149. doi: 10.3390/s26196149 (PMC13667481; doi:10.3390/s26196149)
Supplement: Supplementary file 1 [file sensors-26-06149-s001.zip › sensors-4490198-supplementary.pdf]

Supporting Information

Dual-Site Synergistic Modulation of $La_{0.9}Fe_{0.8}Co_{0.2}O_3$ Perovskite Oxide for High-Performance Methane Sensing in Oil and Gas Pipelines

Lichao Dong [1,2], Zhenzhu Xi [1,*], Lu Hu [2] and Jiangang Zheng [2]

[1] State Key Laboratory of Critical Minerals, School of Geosciences and Info-Physics, Central South University, Changsha 410083, China; dlc_cq@petrochina.com.cn
[2] No.12 Oil Production Plant of Changqing Oilfield, Qingyang 745400, China; hul_cq@petrochina.com.cn (L.H.); zjig_cq@petrochina.com.cn (J.Z.)
* Correspondence: xizhenzhu@csu.edu.cn

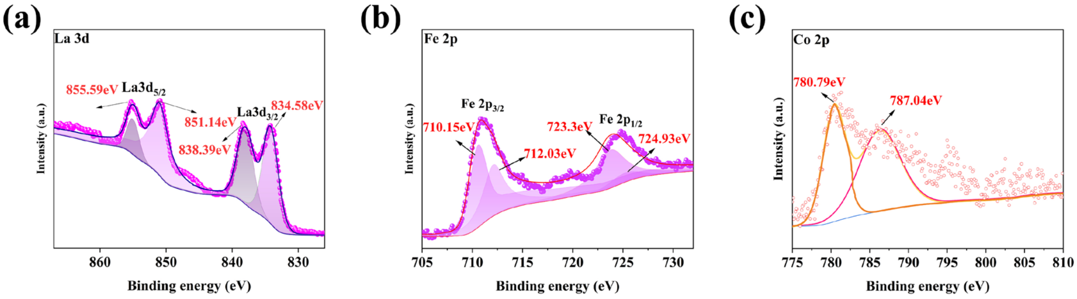

Figure S1. (a) La 3d; (b) Fe 2p; (c) Co 2p comparisons of $La_{0.9}Fe_{0.8}Co_{0.2}O_3$.

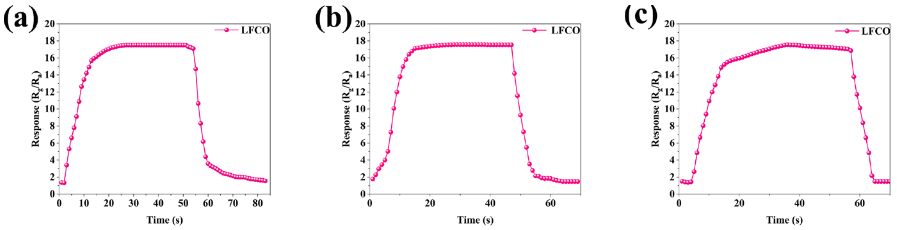

Figure S2. Reproducibility of the LFCO sensor.

Table S1. Comparison of CH_4-sensing performance of gas sensors.

Materials	Temperature (°C)	Concentration (ppm)	Response/ Sensitivity	Response time (s)	Recovery time (s)	Ref
Pd@Ni/ZnO	60	5000	904.04	4	174	[1]
1.5 at.% Ag/ ZnO	200	5000	20.15	118	119	[2]
0.5 wt% Cr-SnO_2	350	250	79.7	3.9	-	[3]
Ag/CeO_2-CuO Nanorods	175	10000	3	23	27	[4]
ZnO-xCo_3O_4	300	400	$<$5	-	-	[5]
TiO1.5/ZnO nanocomposites	300	1000	1.201	430	228	[6]
$La_{0.9}Fe_{0.8}Co_{0.2}O_3$	125	100	17.55	9.9	7.2	This work

1. Sun, X.; Tang, M.; Yu, M.; Fan, Y.; Qin, C.; Cao, J.; Wang, Y. UV-activated CH_4 gas sensor based on Pd@Ni/ZnO microspheres. Materials Today Communications 2024, 40.

2. Wang, Y.; Cui, Y.; Meng, X.; Zhang, Z.; Cao, J. A gas sensor based on Ag-modified ZnO flower-like microspheres: Temperature-modulated dual selectivity to CO and CH_4. Surfaces and Interfaces 2021, 24.

3. Bunpang, K.; Wisitsoraat, A.; Tuantranont, A.; Singkammo, S.; Phanichphant, S.; Liewhiran, C. Highly selective and sensitive CH_4 gas sensors based on flame-spray-made Cr-doped SnO_2 particulate films. Sensors and Actuators B: Chemical 2019, 291, 177-191.

4. Pholoana, M.G.; Malgas, G.F.; Morulane, K.L.; Tshilongo, J.; Swart, H.C.; Motaung, D.E. Dual selective sensing of CH_4 and ultra-low NO gas utilizing Ag-decorated CeO_2-CuO nanorods: Role of humidity in p-n conductivity transition. Inorganic Chemistry Communications 2026, 185.
5. Yin, X.-T.; Zhou, W.-D.; Dastan, D.; Li, J.; Tan, X.-M.; Liu, Y.; Gao, X.-C.; Ma, X.-G. Selectivity sensing response of ZnO-xCo_3O_4 based sensor to CO against CH_4. Materials Science in Semiconductor Processing 2022, 149.
6. Vatandoust, L.; Habibi, A.; Naghshara, H.; Aref, S.M. Fabrication and investigation of TiO1.5/ZnO nanocomposite nanosensor for detection of CO and CH_4 gases. Surfaces and Interfaces 2022, 31.
